# Effectiveness of interventions for the remediation of lead-contaminated soil to prevent or reduce lead exposure - A systematic review

**DOI:** 10.1016/j.scitotenv.2021.150480

**Published:** 2022-02-01

**Authors:** Andreea-Iulia Dobrescu, Agnes Ebenberger, Julia Harlfinger, Ursula Griebler, Irma Klerings, Barbara Nußbaumer-Streit, Andrea Chapman, Lisa Affengruber, Gerald Gartlehner

**Affiliations:** a Department for Evidence-based Medicine and Evaluation, Danube University Krems, Krems a.d. Donau, Austria; b RTI International, Research Triangle Park, NC, USA

**Keywords:** BLL, blood lead levels, CG, control group, CI, confidence interval, DLL, dust lead levels, IG, intervention group, NR, not reported, ppm, parts per million, SE, standard error, SES, socioeconomic status, SLL, soil lead levels, US$, United States dollars, vs, versus, Lead, Soil remediation, Children, Blood lead level

## Abstract

**Objective:**

To summarize the evidence on the effectiveness of soil remediation to prevent or reduce lead exposure.

**Methods:**

We systematically searched MEDLINE, the Agricultural & Environmental Science Database, Web of Science, and Scopus from 1980 to February 15, 2021. We also performed reference list checking, hand-searched websites, and contacted experts. Eligible studies evaluated the effect of soil remediation to prevent or reduce lead exposure in humans of any age. We screened all records dually; one investigator performed the data extraction; a second checked for completeness and accuracy. Two investigators independently rated the risk of bias of included studies and graded the certainty of evidence. We synthesized findings narratively.

**Results:**

We identified 6614 potentially relevant publications, all focused on children, of which five studies (six records) fulfilled our prespecified inclusion criteria. The number of evaluated participants ranged from 31 to 1425, with follow-up periods of 11 months to one year. The primary soil remediation method was the replacement of the upper layer with clean soil. Outcomes were limited to blood lead levels (BLL), dust lead levels, and soil lead levels. The largest study, a controlled before– after study (*n* = 1425) reported favorable effects of soil remediation compared to no intervention. This finding was consistent with results from two cross-sectional studies and one uncontrolled before–after study. One year post-remediation, the mean reduction in BLL was 2.1 μg/dL (*p* < 0.0001) greater in the intervention group than in the control group. Two randomized controlled trials with a total of 511 participants showed no statistically significant incremental effect of soil remediation when combined with paint and/or dust abatement. The certainty of evidence for all outcomes was low.

**Conclusion:**

Soil remediation appears to reduce BLL in children when used as a single intervention. The incremental benefit of soil remediation when part of other interventions is limited.

## Highlights

• Lead exposure from contaminated soil is an important public health issue, concerning both children and adults.
• Soil remediation alone or in combination with other interventions appears to reduce the blood lead levels in children.
• The effect of soil remediation on health outcomes, in high lead exposure areas or in low/middle-income countries is unclear.

## Graphical abstract

**Figure:**
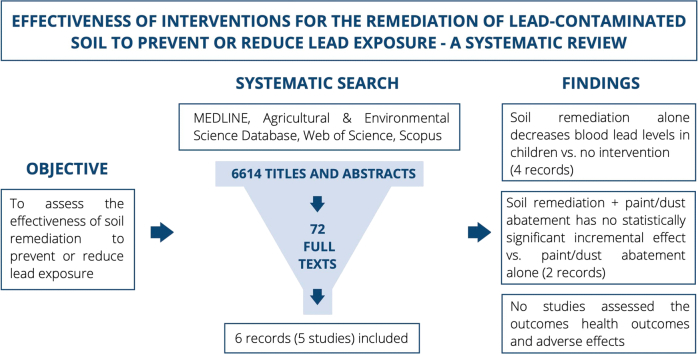

## 1 Introduction

The World Health Organization (WHO) has designated lead as one of ten chemicals of major public health concern (World Health Organization, 2018). Lead can cause acute and chronic illnesses of various organ systems, affecting both children and adults. Chronic lead poisoning is more common than acute poisoning. In adults, it typically leads to memory and concentration problems, depression, abdominal and neuromuscular symptoms, fatigue, anemia, sleep disturbance, hypertension, and cardiovascular diseases (Lanphear et al., 2018). In children who are chronically exposed to lead, aggressive behaviour and apathy are the most common symptoms (Pearce, 2007; Patrick, 2006). No level of lead exposure has so far been identified that is without harmful effects (World Health Organization, 2018; Bellinger, 2008). Blood lead levels (BLL) lower than 5 μg/dL were associated with reduced school performances in children (Bellinger, 2008). In adults, BLL of 1 μg/dL were associated with an increased risk of cardiovascular diseases (Lanphear et al., 2018). During pregnancy, both current lead exposure and accrued lead in the mother's bones may harm the developing fetus, which may result in miscarriage, stillbirth, premature birth, and low birth weight (World Health Organization, 2018). The harmful effects of lead exposure, however, are preventable (World Health Organization, 2018). Therefore, the identification and control of lead hazards in residential environments are vital (Chisolm, 2001). Lead exposure can come from various sources (e.g. lead mines, smelters, refineries, recycling and manufacturing sites, leaded petrol and aviation fuel, lead-based paint, water piping, fixtures and solder). Contaminated soil is often an important source of lead exposure (Boreland and Lyle, 2014; von Lindern et al., 2003a; Tirima et al., 2016) for children because it also accumulates as indoor dust (World Health Organization, 2018; Laidlaw et al., 2017). Compared with adults, children have a higher exposure to lead-contaminated soil and indoor dust, as they place hands and objects in their mouths and are closer to the ground, for instance due to crawling and playing. In addition, the absorption and retention of lead is higher in children than in older individuals (World Health Organization, 2018; Bellinger, 2008; Lanphear et al., 1998). Lead can pass the developing blood-brain barrier, making children especially vulnerable because their nervous system is still developing (Mason et al., 2014).

The Center for Disease Control and Prevention (CDC) recommends taking various management actions for children with BLL greater or equal to 5 μg/dL [0.24 μmol/L] (Advisory Committee for Childhood Lead Poisonning Prevention, 2012). As reported in a narrative review from 2017, in some urban neighborhoods in the United States (US), up to 20–40% of children had elevated BLL (≥5 μg/dL), which were, at least partly, attributed to lead in soil (Laidlaw et al., 2017). According to the US Environmental Protection Agency, the acceptable safety standards for lead in bare soil are under 400 ppm (ppm) in children's play areas and under 1200 ppm for bare soil in non-play areas (Agency for Toxic Substances and Disease Registry, 2019).

The remediation of lead-contaminated soil aims to minimize or eliminate the hazard, and a range of soil remediation techniques exists. These approaches, some of which are still experimental, include mechanical, chemical, or biological interventions to clean, stabilize, remove, replace, and/or cover contaminated soil (Laidlaw et al., 2017).

To date, the effectiveness of soil remediation to reduce the negative health effects of lead exposure has not been assessed comprehensively and systematically. A narrative review including soil remediation studies with various techniques (excavation and replacement) to prevent or reduce soil contamination in urban environments reported a BLL decrease that ranged from 35% to 90% after six months to three years post-remediation (Laidlaw et al., 2017). A Cochrane review published in 2020 that assessed the effects of dust abatement in households with high lead exposure, included two studies on the remediation of contaminated soil with insufficient evidence on the effectiveness of soil remediation (Nussbaumer-Streit et al., 2020).

The aim of our review was to support WHO to develop guidelines and systematically assess the effects of soil remediation interventions on human health in rural and urban environments. Specifically, we strove to answer the following research question:

- What are the possible effects and/or adverse effects of soil remediation, alone or in combination with other interventions, compared to no interventions or other interventions on BLL and the subsequent health outcomes in humans?

## 2 Methods

This systematic review was conducted in accordance with Cochrane systematic review methods (Higgins and Thomas, 2019) Throughout the manuscript, we followed the Preferred Reporting Items for Systematic Review and Meta-Analysis Protocols (PRISMA) statement (Moher et al., 2009).

We registered the protocol in PROSPERO (the International Prospective Register of Systematic Reviews), ID CRD42019136676.

### 2.1 Search strategy and criteria

An experienced information specialist searched Ovid MEDLINE, the Agricultural & Environmental Science Database (ProQuest), the Web of Science (Science Citation Index Expanded [SCI-EXPANDED], Social Sciences Citation Index [SSCI], Clarivate), and Scopus (Elsevier) from 1980 to February 15, 2021.

The searches combined free-text search terms and controlled vocabulary (e.g., Medical Subject Headings [MeSH]) if available and were limited to English, German, or French language records. The full search strategies are reported in Appendix A.

Further, we searched the bibliographies and reference lists of selected publications for relevant citations that were missed by our database searches. We also sought information about eligible studies via correspondence with the WHO Guideline Development Panel and WHO technical experts. To identify study reports not published in scientific journals, we manually websites from the following governmental and nongovernmental agencies: the US Department of Housing and Urban Development (up to 2019), the Australian National Health and Medical Research Council, Health Canada, Médecins sans Frontières, the National Service Center for Environmental Publications, and the WHO International Clinical Trials Registry Platform (ICTRP) Search Portal. The web search was carried out in July 2019 and updated in April 2021. The search terms we used are reported in Appendix A.

### 2.2 Eligibility criteria

We were interested in any study designs that evaluated different soil interventions with the aim to prevent or reduce lead exposure. Table 1 presents the a priori–defined eligibility criteria.

**Table 1:** Eligibility criteria.

	Inclusion	Exclusion
Population	People of any age exposed to lead from contaminated soil: - Children, including infants - Adolescents - Pregnant women (as surrogates for unborn children) - General adult population	- People who are occupationally exposed to lead - Animals and plants - Studies not assessing humans (e.g., only soil or dust)
Intervention	Interventions that treat soil with the aim to prevent or reduce lead exposure in humans, including: - Physical remediation (e.g., removal, excavation, replacement, surface capping, encapsulation, solidification of soil) - Chemical remediation (e.g., calcium phosphate, biochar, Maectite®, soil washing, stabilizing agents) - Biological remediation (e.g., phytoremediation, fungal remediation, microbial remediation) - Thermal remediation (e.g., vitrification) - Combinations of these interventions	- Combinations of soil remediation interventions with additional interventions not involving soil unless the incremental effect of the soil remediation is estimated individually - Interventions to reduce lead exposure from sources other than soil (e.g., paint, drinking water) - Educational interventions - Interventions to reduce occupational lead exposure
Comparison	- No intervention - Other interventions (e.g., educational) addressing soil lead exposure - Combinations of soil remediation interventions and other interventions not involving soil	- Interventions that aim to reduce lead exposure from sources other than soil (paint, drinking water, consumer products, etc.), where the effects of the soil remediation component cannot be estimated separately
Outcomes	*Exposure outcomes:* - BLL - Soil or dust lead levels if reported together with outcomes in humans *Health outcomes:* - Acute or chronic lead poisoning - Cognitive and neurobehavioral outcomes, particularly in children (standardized intelligence quotient, behavioral and developmental measures) - Physical development in children (standardized motor skill measures) - Adverse pregnancy outcomes - Cardiovascular outcomes - Renal outcomes - Fertility outcomes - Anemia, hemoglobin levels *Other outcomes:* - Adverse effects of the intervention - Costs of intervention if reported additionally to outcomes in humans	- Exclusively nonhuman outcomes such as lead concentration in soil, plants, or animals
Timing	- Studies published from 1980 onward - Any follow-up duration	- Studies published before 1980
Setting	- Any rural or urban settings with lead-contaminated soil - Settings with and without ongoing lead deposition (e.g., active smelters)	
Study designs	- RCTs - Cluster RCTs - Non-RCTs - ITS studies - Before–after studies - Prospective cohort studies - Repeated cross-sectional studies	- Single time point cross-sectional studies - Case–control studies - Ecological studies - Economic studies - Modeling studies/in vitro studies - Narrative reviews - Systematic reviews
Document types	- Journal articles published in peer-reviewed journals - Reports, theses	- Study registry entries with/without results - Conference abstracts - Research/study protocols - Posters - Books or book chapters
Languages	- English, German, French	- Other languages

*Abbreviations:* BLL, blood lead levels; e.g., for example; ITS, interrupted time series; RCT(s), randomized controlled trial(s).

### 2.3 Study selection and data extraction

Abstract and full-text review forms were developed and piloted on a sample of 50 abstracts and five full-text articles by all reviewers, working in pairs. Discrepancies were resolved by discussion or by involving a third reviewer. During the study selection process, the abstracts and selected full-text articles were independently reviewed by two investigators using Covidence (Covidence systematic review software, n.d.). After pilot-testing the data extraction forms, one investigator extracted data from the included studies; a second investigator checked for completeness and accuracy. We extracted relevant information related to the characteristics of the study populations, settings, interventions, comparators, study designs, methods, outcomes of interest, and results. We also abstracted data on soil and dust lead levels as well as soil remediation costs

### 2.4 Data synthesis

We summarized the results narratively and grouped them by outcomes of interest. We did not identify enough studies with a similar design to be able to conduct meta-analyses.

### 2.5 Risk of Bias assessment

Two reviewers independently assessed the risk of bias of the included studies. Disagreements were resolved by discussion and consensus or by consulting a third reviewer. We used the Cochrane Risk of Bias Tool 2.0 (RoB2) for assessing the quality of individually randomized controlled trials (RCTs) (Sterne et al., 2019) and a previous version of the tool that is compatible with cluster RCTs (Lyu et al., 2018). For nonrandomized studies that met the Effective Practice and Organization of Care (EPOC) criteria, we used the Cochrane EPOC risk of bias tool (Effective Practice and Organisation of Care Group (EPOC), 2017). For studies that did not meet the EPOC criteria (Cochrane Effective Practice and Organisation of Care (EPOC), 2017), we assessed the risk of bias using the Quality Assessment Tool for Quantitative Studies, developed by the Effective Public Health Practice Project (EPHPP) (Effective Public Health Practice Project, n.d.).

For the RoB2 and EPOC risk of bias criteria, we rated the risk of bias using the categories “low,””some concerns,” and “high.” For the EPHPP, the categories were “strong,” “moderate,” and “weak.” Weak corresponded to a high risk of bias, moderate to some concerns, and strong to a low risk of bias.

### 2.6 Certainty of evidence

We dually rated the certainty of evidence for the outcomes ranked as critical or important by the WHO Guideline Development Group using the Grading of Recommendations Assessment, Development and Evaluation (GRADE) approach (Guyatt et al., 2011). These ratings incorporate assessments of the risk of bias, inconsistency, indirectness, imprecision, and publication bias for each outcome. Depending on the certainty of evidence, the overall rating for each outcome resulted in one of four categories: high, moderate, low, or very low. Disagreements were resolved by discussion.

### 2.7 Role of the funding source

This review was funded by a contract with WHO. The WHO Guideline Development Committee assisted in the development of the key questions, study inclusion criteria, and outcome measures of interest but was not involved in the data collection, analysis, or manuscript preparation.

## 3 Results

### 3.1 Literature search results

**Fig. 1:**
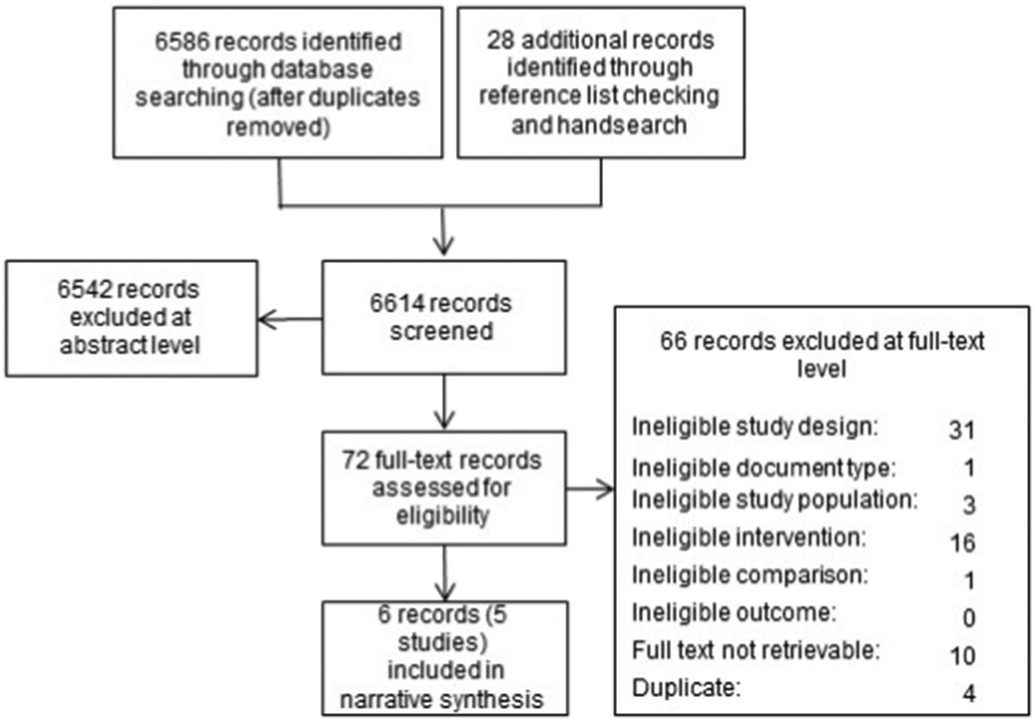
Selection of the included studies.

Our literature searches detected 6614 unique records after deduplication. We retrieved 72 as full-text publications. Five studies corresponding to six publications met our eligibility criteria (Aschengrau et al., 1994; Weitzman et al., 1993; Farrell et al., 1998; von Lindern et al., 2003b; Lanphear et al., 2003; Gagne, 1994). One study reported controlled short-term and uncontrolled long-term findings of one study arm (Aschengrau et al., 1994; Weitzman et al., 1993). Due to the different study designs, number of participants, and additional interventions during the long-term phase, we present the results of these two publications separately. Fig. 1 depicts the literature review flow. Appendix B lists the studies excluded at the full-text level and the reasons for exclusion.

In the following sections, we first summarize the characteristics of the included studies (Table 2). We then present the effect of soil remediation on the outcomes of interest (Table 3). All the included studies assessed BLL. The costs associated with soil remediation were reported in three studies (Weitzman et al., 1993; Farrell et al., 1998; Gagne, 1994). None of the included studies reported on health outcomes or adverse effects of the intervention.

**Table 2:** Characteristics of the included studies.

Study	Study design	Population (n)	Participant characteristics	Location; source of contamination	Intervention	Comparison	Risk of bias
Aschengrau, 1994 (Aschengrau et al., 1994)	uCBA	31 IG1 = 18 IG2 = 13	NR	Boston, USA; no point source/smelter (potentially multiple sources such as exterior paint, past deposition of airborne lead from gasoline)	*Soil intervention:* 15 cm topsoil removed from entire yard, replaced with 20 cm of clean soil; water-permeable geotextile fabric barrier used to protect against recontamination; surface soil covered with either sod, grass seeding, bark, or mulch *Co-intervention*^b^: interior dust abatement and loose paint stabilization in IG1; paint stabilization alone in IG2	Interior dust abatement and loose paint stabilization in IG1; paint stabilization alone in IG2	High
Farrell, 1998 (Farrell et al., 1998)	Cluster RCT, by neighborhood	408 IG: 212 CG: 196	Age range: 6 mo.–6 yrs. Sex^c^: NR Ethnicity^d^: NR	Baltimore, USA; lead-based paint as main source	*Soil intervention*: soil was remediated when one surface sample showed average SLL > 500 ppm; removal of the top 15 cm of soil, replacing it with “lead-free” soil (less than 50 ppm), and then sodding or seeding *Co-intervention:* Exterior paint stabilization	Exterior paint stabilization only	High
Gagné, 1994 (Gagne, 1994)	Repeated cross-sectional study	117	Age range: 1–5 yrs. Sex^c^: NR Ethnicity^d^: NR	Notre-Dame district of Rouyn-Noranda, Quebec, Canada; active copper smelter	*Soil intervention*: removal of first 10 cm of yard soil (exceeding 500 ppm), replacement with uncontaminated soil and grass on top (or gravel in parking areas) *Co-intervention*: NA	No intervention	High
Lanphear,2003 (Lanphear et al., 2003)	Repeated cross-sectional study with CG	Time point 1 (1989): 112 (73 IG, 39 CG) Time point 2 (1998): 198 (167 IG, 31 CG)	Age range: 0.5–12 yrs. Sex^c^: NR Ethnicity^d^: NR	Midvale, Utah, USA; smelter and milling	*Soil intervention:* remediation of yards with average SLL > 500 ppm (1993–1996): excavation to a depth of 18 in. (46 cm) and backfill with clean soil Clay cap over the tailings at the former mining and milling site (1993) *Co-intervention*: NA	No intervention	Some concerns
von Lindern, 2003 (von Lindern et al., 2003b)	CBA	1425 IG: 238 CG: 1187	Age range: < 9 yrs. Sex^c^: NR Ethnicity^d^: NR	Bunker Hill Superfund Site, Idaho, USA; abandoned industrial complex for lead-zinc mining and smelting	*Soil intervention*: replacement of contaminated surface soils and dusts with clean dirt in all yards having SLL > 1000 ppm *Co-intervention*: NA	No intervention	High
Weitzman, 1993 (Weitzman et al., 1993)	RCT	103^a^ IG: 52 CG: 51	Mean age at baseline: - overall: 31.6 mo. - IG: 30.5 mo. - CG: 31.4 mo. Sex^c^: - Overall: 53.3% - IG: 59.6% - CG: 49% Ethnicity/Race^d^: Overall: 93% IG: 92% CG: 92%	Boston, USA; no point source/smelter (potentially multiple sources such as exterior paint, past deposition of airborne lead from gasoline)	*Soil intervention:* 15 cm topsoil removed from entire yard, replaced with 20 cm of clean soil; water-permeable geotextile fabric barrier used to protect against recontamination; surface soil covered with either sod, grass seeding, bark, or mulch) *Co-intervention*: loose interior paint removal, interior dust abatement	Interior dust abatement and loose interior paint removal only	Some concerns

*Abbreviations*: CBA, controlled before–after study; CG, control group; cm, centimeter; e.g., for example; IG, intervention group; mo., month; n, population number; NA, not applicable; NR, not reported; ppm, parts per million; RCT, randomized controlled trial; SLL, soil lead levels; uCBA, uncontrolled before–after study; USA, United States of America; yrs., years.

a We only used data from the study group and control group A. Control group B is not relevant for our analysis because the interventions that its participants received differed in more respects than only soil remediation.

b The co-interventions were performed during phase one of the study.

c Percentage of males.

d Non-white population.

**Table 3:** Summary of the baseline blood lead levels and results of the included studies' interventions.

First author, year	BLL at baseline (μg/dL)	Decrease in BLL (μg/dL)	Cost (US$)^a^	Soil/dust lead levels
Aschengrau, 1994 (Aschengrau et al., 1994)	IG1: 12.94 IG2: 10.54	IG1: - 5.24 IG2: - 2.57	NR	*DLL:* mean floor lead levels were unchanged at 6–12 months after the soil remediation intervention in both evaluated groups (*p* = 0.95 and 0.15, respectively) *SLL:* IG1: 93% reduction (from 2358 ppm to 171 ppm) IG2: 92% reduction (from 2299 ppm to 180 ppm)
Farrell, 1998 (Farrell et al., 1998)	IG (geometric mean [95% CI]): 11.0 (4.7, 23.0) CG: 10.9 (4.1, 29.1) *Subjects who completed the study:* IG (geometric mean [95% CI]): 12.1 (5.5, 22.0) CG (geometric mean [95% CI]): 10.9 (4.1, 23.5)	Difference between the IG and CG: −0.05 (SE 0.037), 95% CI: (−0.12, 0.03)	2163	*DLL*: NR *SLL*: 93% reduction in the intervention area (from 503.6 ppm to 33.6 ppm)
Gagné, 1994 (Gagne, 1994)	*1989*: 10 (NR) (95th percentile 20 μg/dL)	*Geometric mean BLL:* 1991 (after intervention): 7.3 (95th percentile: 14.7 μg/dL) *- Pre-post mean difference 1989–1991*: −2.7 BLL greater than 10 μg/dL: ca. 100% in 1979, ca. 50% in 1989, ca. 25% in 1991	5000	*DLL*: NR *SLL*: NR
Lanphear, 2003 (Lanphear et al., 2003)	IG: 5.6 (95% CI: 4.9–6.3) CG: 3.9 (95% CI: 3.2–4.7)	*Difference in decrease in mean BLL IG* vs. *CG*: - age between 6 and 72 months: 2.3, 95% CI: (1.8, 2.9), *p* = 0.14 - age between 6 and 36 months: 2.5, 95% CI: (1.8; 3.5), *p* = 0.03	NR	*DLL* (absolute difference): 252 μg/g, *p* = 0.0005 *SLL* (absolute difference): 439 μg/g, *p* = 0.0001
von Lindern, 2003 (von Lindern et al., 2003b)	*Prior to 1990 remediation:* IG: 15.3 (NR) CG: 10.2 (NR) *Prior to 1998 remediation:* IG: 7.3 (NR) CG: 4.8 (NR)	*Difference in mean reduction IG* vs. *CG*: −2.1, *p* < 0.0001 (95% CI NR) *The mean difference in BLL from baseline to follow-up:* IG: −2.5 (95% CI NR), p < 0.0001 CG: −0.4 (95% CI NR), p < 0.0001	NR	*DLL:* NR *SLL* (yard): 88% reduction (from 1700 ppm to 200 ppm)
Weitzman, 1993 (Weitzman et al., 1993)	IG: 13.1 (NR) CG: 12.4 (NR)	*Adjusted difference (baseline BLL, age, race and ethnicity, household members exposed to lead at work)* IG vs. CG: 0.90 (95% CI: 0.23, −2.04) *Adjusted difference (baseline BLL, race and ethnicity, SES, playing or sitting on the floor) IG* vs. *CG*: 0.80 (95% CI: 0.45, −2.05)	9600	*DLL:* IG: 53% reduction CG: 49% reduction *SLL:* Average decrease of 1790 ppm (range between 160 ppm and 5360 ppm)

**Abbreviations:** BLL blood lead levels; CG, control group; CI, confidence interval; DLL, dust lead levels; IG, intervention group; NR, not reported; ppm, parts per million; SE, standard error; SES, socioeconomic status; SLL soil lead levels; US$, United States dollars; vs. = versus.

a Average cost of soil remediation per property/lot, otherwise mentioned.

### 3.2 Characteristics of the included studies

Five studies (six publications) met our inclusion criteria (Aschengrau et al., 1994; Weitzman et al., 1993; Farrell et al., 1998; von Lindern et al., 2003b; Lanphear et al., 2003; Gagne, 1994); four were conducted in the US (Aschengrau et al., 1994; Weitzman et al., 1993; Farrell et al., 1998; von Lindern et al., 2003b; Lanphear et al., 2003) and one in Canada (Gagne, 1994) between 1988 (von Lindern et al., 2003b) and 1996 (Lanphear et al., 2003). Four studies assessed the effectiveness of soil remediation in urban areas (Aschengrau et al., 1994; Weitzman et al., 1993; Farrell et al., 1998; Lanphear et al., 2003; Gagne, 1994) and one in a rural setting (von Lindern et al., 2003b). The sources of soil lead contamination were nearby mines and/or smelters (von Lindern et al., 2003b; Lanphear et al., 2003; Gagne, 1994) and lead-based paint (Aschengrau et al., 1994; Weitzman et al., 1993; Farrell et al., 1998). The study designs and methodological approaches included one cluster-randomized trial at the neighborhood level (Farrell et al., 1998), one individually randomized trial (Weitzman et al., 1993), and one uncontrolled before–after study design (Aschengrau et al., 1994) belonging to the same study, one controlled before–after study (von Lindern et al., 2003b), one repeated cross-sectional study with a control group (Lanphear et al., 2003), and one repeated cross-sectional study without a control (Gagne, 1994).

All studies focused on children, aged between 6 months (Farrell et al., 1998) and 12 years (Lanphear et al., 2003) with BLL at baseline from 5.6 μg/dL (Lanphear et al., 2003) to 15 μg/dL (von Lindern et al., 2003b) in the intervention groups. The number of participants ranged from 31 (Aschengrau et al., 1994) to 1425 (von Lindern et al., 2003b). Specific soil remediation interventions involved the removal of 10 cm (Gagne, 1994), 15 cm (Aschengrau et al., 1994; Weitzman et al., 1993; Farrell et al., 1998), or 46 cm (Lanphear et al., 2003) of the upper layer of contaminated soil and replacement with clean soil. In one study (von Lindern et al., 2003b), all yards having soil lead concentrations greater than 1000 mg/kg were identified and replaced. All studies remediated residential lots; in addition, some focused on public areas such as parks and parking lots (von Lindern et al., 2003b; Gagne, 1994). Three studies (Aschengrau et al., 1994; Weitzman et al., 1993; Farrell et al., 1998; Gagne, 1994) explicitly reported that sod/grass or other materials were placed on top of the clean soil. Two studies performed other interventions in addition to the soil remediation: a one-time interior dust abatement and loose paint stabilization (Aschengrau et al., 1994; Weitzman et al., 1993), and exterior paint stabilization (Farrell et al., 1998).

### 3.3 Effect of soil remediation on blood Lead levels

All studies assessed the effect of soil remediation on BLL in children. Table 3 summarizes the main results. Appendix C presents the certainty of evidence ratings for each outcome.

#### 3.3.1 Soil remediation versus no intervention

Three studies assessed the effects of soil remediation compared with no intervention (von Lindern et al., 2003b; Lanphear et al., 2003; Gagne, 1994). One controlled before–after study with a high risk of bias compared pre-remediation to one-year post-remediation BLL in 1425 children under the age of 9 years living near an abandoned industrial lead-zinc mining and smelting complex in the Bunker Hill Superfund Site, Idaho, USA (von Lindern et al., 2003b). The investigators recruited participants over the course of 10 years and grouped them into 10 intervention and control groups. The soil remediation intervention consisted of replacing contaminated surface soil and dust with uncontaminated soil in yards having soil lead levels greater than 1000 ppm. A statistically significantly greater mean reduction in BLL of −2.1 μg/dL (95% CI NR) (*p* < 0.0001) was observed in the intervention group compared to the control one year post-remediation.

One repeated cross-sectional study with a control group, rated as having some methodological concerns, assessed the effect of the remediation of yards with average soil lead levels greater than 500 ppm in Midvale, Utah, USA (Lanphear et al., 2003). The intervention consisted in excavation and backfill with clean soil. Using an identical protocol, the authors evaluated two distinct cohorts of children between 6 months and 12 years at two time points 10 years apart (Lanphear et al., 2003). No baseline characteristics of the two groups (from 1989 and 1998) were provided. After adjustment for covariates (age, mouthing behaviour, year of study, socioeconomic status), the results indicated no significant reduction in BLL between children, aged 6 and 72 months, who lived in abated and non-abated houses. In contrast, a significant decline in BLL was reported for children from 6 to 36 months old who had not been exposed to lead contaminated yards in early childhood (absolute decline in blood lead concentration 2.5 μg/dL; *p* = 0.03).

An uncontrolled repeated cross-sectional study by Gagné et al. measured the BLL in 233 children from a residential area close to an active copper smelter in Quebec, Canada (Gagne, 1994). Before the soil remediation, the geometric mean BLLs of the selected group were 10 μg/dL (95th percentile: 20 μg/dL). The soil remediation (1990–1991) consisted of removing the first 10 cm of soil and replacing it with uncontaminated soil and grass on top (or gravel in parking areas). By the time the last lot was remediated in 1991, the mean BLL decreased to 7.3 μg/dL (*p*-value not reported). The proportion of children with BLL greater than 10 μg/dL decreased from almost 100% in 1979 to 50% in 1989 and finally to 25% in 1991. No potential confounders were considered. It is not clear which portion can be attributed to soil remediation because of concomitant lead reduction programs (reduction of stark emissions and ban of lead from gasoline) (Gagne, 1994).

We rated the certainty of evidence as low that soil remediation compared with no intervention reduces the BLL in children (Appendix C).

#### 3.3.2 Soil remediation with co-interventions versus co-interventions alone

Two studies (three publications) compared soil remediation plus other interventions versus other interventions alone and provided findings about the incremental benefits of soil remediation when used in combination with other lead-reducing interventions (Aschengrau et al., 1994; Weitzman et al., 1993; Farrell et al., 1998).

A cluster RCT by Farrell et al., which evaluated 408 children over 6 months, randomized contaminated neighborhoods to exterior paint stabilization with or without the replacement of 15 cm of soil (Farrell et al., 1998). We rated the study as having a high risk of bias because of the high attrition (55%). The geometric mean BLL of participants who completed the study decreased from 12.1 μg/dL to 9.7 μg/dL due to soil remediation. After adjustment for covariates (time, seasonality, socioeconomic status, age, and mouthing behaviour), the authors reported no statistically significant difference between the intervention and the control group (covariate-adjusted effect estimate: −0.05 μg/dL [standard error: 0.04]).

The second study, the Boston project, included children between 6 months and 5 years exposed to lead, and assessed the effect of soil remediation in contaminated areas. Phase one of the project was an RCT reported by Weitzman et al. (Weitzman et al., 1993) The interventions consisted of loose interior paint removal and interior dust abatement with and without the replacement of 15 cm of contaminated soil (Weitzman et al., 1993). We rated the study as having some methodological concerns. After 11 months, the mean BLL of the intervention group including soil remediation decreased by 1.53 μg/dL more than that of the comparison group (95% CI: −2.87, −0.19 μg/dL). When confounding variables were adjusted for (race and ethnicity, socioeconomic status, playing or sitting on the floor, and baseline BLL), the adjusted mean decrease in BLL was −0.80 μg/dL (95% CI: −2.05 to 0.45).

Using an uncontrolled before–after study design, Aschengrau et al. reported the results of the second phase of the Boston study (Aschengrau et al., 1994). Participants who received paint stabilization and dust abatement or dust abatement alone during phase one received soil remediation in phase two. The study was rated as having a high risk of bias because of the study design and a high dropout rate. When the analysis was restricted for the season or to only one child from each family and adjusted for baseline BLL, the decrease in BLL after the soil remediation was −5.24 μg/dL and − 2.57 μg/dL, respectively (Aschengrau et al., 1994).

We rated the certainty of evidence as low that soil remediation when added to paint stabilization and dust abatement leads to a small incremental reduction in BLL in children (Appendix C).

### 3.4 Effect of soil remediation on health outcomes

No evidence on patient-relevant health outcomes was identified.

### 3.5 Soil lead levels

Two RCTs (Weitzman et al., 1993; Farrell et al., 1998), two before–after studies (one with a control group) (Aschengrau et al., 1994; von Lindern et al., 2003b), and one repeated cross-sectional study (Lanphear et al., 2003) assessed the effect of soil remediation on soil lead levels. All reported a statistically significant reduction after the intervention.

In three publications, the reduction percentage ranged from 88% (von Lindern et al., 2003b) to 93% (Aschengrau et al., 1994; Farrell et al., 1998). Weitzman et al. reported an average decrease of 1790 ppm (range between 160 ppm and 5360 ppm) (Weitzman et al., 1993) while Lanphear et al. reported an absolute difference of 439 ppm (*p* = 0.0001), between the abated and nonabated yards (Lanphear et al., 2003).

Two RCTs and one uncontrolled before–after study also assessed recontamination (Aschengrau et al., 1994; Weitzman et al., 1993; Farrell et al., 1998). One RCT reported no statistically significant increase in soil lead levels 9 months post-remediation (Weitzman et al., 1993); the other RCT mentioned “significant lead reaccumulation” (no data available) two years post-remediation (Farrell et al., 1998). Aschengrau et al. reported that 44% of the remediated yards from the first intervention group and 62% from the second intervention group showed evidence of recontamination at 6–10 months post remediation (Aschengrau et al., 1994).

### 3.6 Dust lead levels

Two studies (three publications) reported floor dust levels and showed a statistically significant decrease after soil remediation (Aschengrau et al., 1994; Weitzman et al., 1993; Lanphear et al., 2003). Weitzman et al. found that 4 to 5 weeks after phase one of the Boston project, the median floor dust lead concentrations in the intervention (soil remediation, loose interior paint removal, interior dust abatement) and control groups (loose interior paint removal, interior dust abatement) were reduced by 53% and 49%, respectively (Weitzman et al., 1993). In the second phase of the same project, the mean floor lead levels were unchanged at 6 to 12 months after the soil remediation intervention in both groups, compared with baseline levels (Aschengrau et al., 1994).

Lanphear et al. reported a statistically significant reduction in the floor dust lead level in the soil remediation group (geometric mean decrease: 409 μg/dL) compared with the control group (geometric mean decrease: 157 μg/dL), *p* < 0.001 (Lanphear et al., 2003).

### 3.7 Adverse events of soil remediation

None of the included studies reported on adverse effects of the soil remediation intervention.

### 3.8 Cost of soil remediation

Three studies reported the cost of the soil remediation (Weitzman et al., 1993; Farrell et al., 1998; Gagne, 1994). The costs per remediated property varied from US$ 2163 (1989–1990) (Farrell et al., 1998), US$ 5000 (1990–1991) (Gagne, 1994) to US$ 9600 (1989–1990) (Weitzman et al., 1993).

## 4 Discussion

To our knowledge, this is the first systematic review on the effectiveness of soil remediation to prevent or reduce lead exposure and, subsequently, to avoid or mitigate negative health effects in humans of all ages.

We identified five eligible studies (Aschengrau et al., 1994; Weitzman et al., 1993; Farrell et al., 1998; von Lindern et al., 2003b; Lanphear et al., 2003; Gagne, 1994), all of which focused on children exposed to lead in urban (Aschengrau et al., 1994; Weitzman et al., 1993; Farrell et al., 1998; Lanphear et al., 2003; Gagne, 1994) or rural areas (von Lindern et al., 2003b). The most recent included studies were published in 2003 (von Lindern et al., 2003b; Lanphear et al., 2003); the others were published before 2000.

Overall, the available evidence is limited in quality and quantity. Findings from four nonrandomized studies suggested favorable effects of soil remediation compared to no intervention regarding BLL in children (Aschengrau et al., 1994; von Lindern et al., 2003b; Lanphear et al., 2003; Gagne, 1994). In addition, they reported statistically significant decreases of soil- and dust-lead levels (Weitzman et al., 1993; Farrell et al., 1998) (Aschengrau et al., 1994; von Lindern et al., 2003b) (Lanphear et al., 2003). The significance of the estimated effect sizes from a clinical and public health perspective, however, is unclear. The incremental benefit of soil remediation when added to other interventions to reduce lead exposure in children was modest. Two RCTs reported no statistically significant incremental effects of soil remediation when combined with paint stabilization or dust abatement (Weitzman et al., 1993; Farrell et al., 1998). Due to limitations in the conduct and design of the studies, we rated the certainty of evidence as low which means that future studies might have a substantial impact on these effect estimates.

We also identified 10 studies, which did not meet our inclusion criteria because they assessed combinations of different interventions including soil remediation (Braun et al., 2018; Boreland et al., 2009; Calder et al., 1994; Chowdhury et al., 2021; Ericson et al., 2018; Goulet et al., 1996; Lalor et al., 2001; Langlois et al., 1996; Maynard et al., 2003; Schoof et al., 2016). Because the effect of the soil remediation could not be isolated, we excluded these studies from our analyses. Results, however, pointed to a potentially greater effect of the combined interventions compared with soil remediation alone.

The internal validity of the included studies is limited, as they all carry at least some concerns for bias, most having a high risk of bias, either inherent to their study design (Lanphear et al., 2003; Gagne, 1994) or due to factors such as low response or completion rates (Farrell et al., 1998) and unaccounted differences between groups at baseline (von Lindern et al., 2003b; Lanphear et al., 2003).

The included studies of our review have several strengths and limitations. Two studies were RCTs (Weitzman et al., 1993; Farrell et al., 1998) and two out of four non-randomized studies included a control group (von Lindern et al., 2003b; Lanphear et al., 2003). One study included more than 1400 participants (von Lindern et al., 2003b). Almost all of the studies adjusted the analyses for potential confounders (Aschengrau et al., 1994; Weitzman et al., 1993; Farrell et al., 1998; Lanphear et al., 2003). Two RCTs and one uncontrolled before–after study assessed soil recontamination (Aschengrau et al., 1994; Weitzman et al., 1993; Farrell et al., 1998).

A notable limitation are the many gaps in reporting, which may be due to the fact that the studies are from the 1990s and early 2000s, when fewer reporting standards existed.

The analysis methods were usually not prespecified and, hence, multiple testing cannot be ruled out. Selective outcome reporting cannot be ruled out, either, when no study protocol exists.

The generalizability of findings may be also compromised by several factors. Four out of five studies evaluated children under 9 years of age, so the results may not be applicable to other groups at particular risk, such as pregnant women as well as adolescents and the general adult population (Aschengrau et al., 1994; Weitzman et al., 1993; Farrell et al., 1998; von Lindern et al., 2003b; Gagne, 1994).

The included studies' BLL were only mildly elevated at baseline, between 5.6 μg/dL (Lanphear et al., 2003) and 15.3 μg/dL (von Lindern et al., 2003b). The effect of soil remediation in high-level exposure could not be assessed.

In three publications, the intervention and control groups received other interventions in addition to soil remediation (paint stabilization and/or dust abatement) (Aschengrau et al., 1994; Weitzman et al., 1993; Farrell et al., 1998); another publication mentioned concomitant lead reduction programs (reduction of stark emissions and ban of lead from gasoline) (Gagne, 1994). Hence, the results showed the added effect of soil remediation, which might not be equal to that of soil remediation alone. Albeit not ideal for a strictly separate effect estimation, this mirrors real-life scenarios. Usually, remediation programs address different sources of exposure (e.g. air, dust, chipping paint, drinking water, consumer products). They are designed to eliminate all possible sources of lead contamination and not primarily to assess and compare the effectiveness of individual interventions.

Finally, the follow-up periods of identical study populations in the included studies did not exceed one year (Weitzman et al., 1993; Farrell et al., 1998; von Lindern et al., 2003b; Gagne, 1994) and all studies were conducted in high income countries. Hence, the generalizability of findings to other settings, such as low- and middle-income countries and the intervention's long-term effects and sustainability cannot be established.

We found no studies that assessed the adverse effects or the effectiveness of soil remediation on patient-relevant health outcomes. The BLL is a surrogate outcome and therefore can only indirectly indicate the health status of an individual. It has limitations because lead stores in bones and can continue to contribute to BLL in the absence of further exposure; post-remediation BLL may still represent past exposures (Laidlaw et al., 2017; Gulson et al., 1996) BLL is hence better suited to measure recent or current exposures at low or moderate levels (Barbosa et al., 2005). Many experts consider BLL the primary biomarker for measuring lead exposure because it has a causal relation with health outcomes (Lanphear et al., 1998; Barbosa et al., 2005; Wang et al., 2015; Schober et al., 2006). New studies focusing on health outcomes are probably not realistic. Considering the complexity of health outcomes (e.g. cognitive and neurobehavioral outcomes, physical development in children, adverse pregnancy outcomes, cardiovascular, renal or fertility outcomes), those studies would need larger sample sizes and a longer follow-up for exposed persons during different stages of life (early childhood, childhood, adolescence, adulthood) to provide valuable results.

Our study has some methodological limitations. Although we applied a vigorous methodology, adhering to the standards provided in the Cochrane Handbook (Higgins and Thomas, 2019), we cannot rule out that we may have missed relevant studies or findings. We limited our eligibility criteria to studies published in English, French and German. A recent systematic review reported a negligible impact on the effect estimates and conclusions of language restrictions for most medical topics (Dobrescu et al., 2021). Nevertheless, we might missed studies published in languages other than English, French and German. While we searched for grey literature, reporting bias is a potential limitation of any systematic review. We were not able to conduct a meta-analysis due to differences in the study designs, BLL at baseline, contamination sources, follow-up times, or sampling methodology.

## 5 Conclusion

Soil remediation appears to reduce BLL in children when used as a single intervention. The incremental benefit of soil remediation, however, is limited, when it is part of other interventions that aim to reduce lead exposure. The quality and quantity of the evidence assessing soil remediation to reduce or prevent lead exposure is limited. No evidence is available to assess the effects of soil remediation on pregnant women or other vulnerable populations, in low- and middle-income countries, and on the health outcomes of exposed individuals. No conclusions about long-term effects are possible.

## Funding

This systematic review was commissioned and funded by the 10.13039/100004423World Health Organization.

## Declaration of competing interest

The authors declare that they have no known competing financial interests or personal relationships that could have appeared to influence the work reported in this paper.

## Appendix A Supplementary data

Supplementary material Image 1
