# Supplementary material for: Effectiveness of interventions for the remediation of lead-contaminated soil to prevent or reduce lead exposure - A systematic review
Source: Sci Total Environ. 2022 Feb 1;806:150480. doi: 10.1016/j.scitotenv.2021.150480 (PMC8655614; doi:10.1016/j.scitotenv.2021.150480)
Supplement: Supplementary file 1 — Supplementary material [file mmc1.docx]

# Appendix A. Search strategies

**Database Searches**

Ovid MEDLINE(R) and Epub Ahead of Print, In-Process & Other Non-Indexed Citations, Daily and Versions(R)

|  | **#** | **Searches** |
| --- | --- | --- |
| A. lead | 1 | exp Lead/ |
|  | 2 | exp Lead poisoning/ |
|  | 3 | Pb.ti. |
|  | 4 | (pb adj1 (concentration? or level? or metal?)).ab. |
|  | 5 | pb.ab. /freq=2 |
|  | 6 | (Lead adj1 (level? or blood or sulphide or sulfide or chloride or chromate or oxide or nitrate or acetate)).ti,ab. |
|  | 7 | (BLL? or B-Pb or lead poison* or lead intoxication or lead toxicity or plumbism or saturnism or colica pictonum).ti,ab. |
|  | 8 | (lead adj2 (expos* or hazard* or pollut* or contaminat* or ingest*)).ti,ab. |
|  | 9 | or/1-8 |
| B. soil | 10 | exp Soil/ |
|  | 11 | Soil Pollutants/ |
|  | 12 | (soil? or sediment? or field? or agricultur* or ground or earth or land or surface?).ti,ab. |
|  | 13 | or/10-12 |
| A+B | 14 | 9 and 13 |
| C. lead contaminated soil | 15 | (lead adj3 (soil? or sediment? or field? or agricultur* or ground or earth or land or surface?)).ti,ab,kf. |
| (A+B) OR C | 16 | 14 or 15 |
| D. Remediation | 17 | Soil Pollutants/ip [Isolation & Purification] |
|  | 18 | Environmental Exposure/pc [Prevention & Control] |
|  | 19 | Environmental Pollution/pc [Prevention & Control] |
|  | 20 | "Environmental Restoration and Remediation"/ |
|  | 21 | Vitrification/ |
|  | 22 | remediation.ti,ab,kf. |
|  | 23 | abatement.ti,ab,kf. |
|  | 24 | (phytoremediation or phytostabili?ation or phytoextraction or phytomediation or phytovolatili?ation).ti,ab,kf. |
|  | 25 | bioremediation.ti,ab,kf. |
|  | 26 | vitrification.ti,ab,kf. |
|  | 27 | ((abate* or abating or clean-up or cleanup or decontaminat* or remedia* biochar?) adj3 (soil? or sediment?)).ti,ab. |
|  | 28 | ((decreas* or reduc* or encapsulat* or extract* or flush* or immobili* or solidifi* or stabili*) adj3 (lead or Pb)).ti,ab. |
|  | 29 | or/17-28 |
| ((A+B) OR C)+D | 30 | 16 and 29 |
| E. remediation of lead contaminated soil | 31 | ((abate* or abating or clean-up or cleanup or remov* or decontaminat* or remedia* or decreas* or reduc* or encapsulat* or barrier? or cutoff wall or extract* or flush* or immobili* or solidifi* or stabili* or "dig and haul" or washing or excavat* or capping or cap or biochar?) adj3 (soil? or sediment? or field? or agricultur* or ground or earth or land or surface?) adj3 (lead or Pb)).ti,ab,kf. |
|  | 32 | ((lead or Pb) adj3 (abate* or abating or clean-up or cleanup or remov* or decontaminat* or remedia* or decreas* or reduc* or encapsulat* or barrier? or cutoff wall or extract* or flush* or immobili* or solidifi* or stabili* or "dig and haul" or washing or excavat* or capping or cap or biochar?) adj3 (soil? or sediment? or field? or agricultur* or ground or earth or land or surface?)).ti,ab,kf. |
|  | 33 | ((abate* or abating or clean-up or cleanup or remov* or decontaminat* or remedia* or decreas* or reduc* or encapsulat* or barrier? or cutoff wall or extract* or flush* or immobili* or solidifi* or stabili* or "dig and haul" or washing or excavat* or capping or cap or biochar?) adj3 (lead or Pb) adj3 (soil? or sediment? or field? or agricultur* or ground or earth or land or surface?)).ti,ab,kf. |
| (((A+B) OR C)+D) OR E | 34 | or/30-33 |
| humans only | 35 | exp animals/ not humans/ |
|  | 36 | 34 not 35 |
| 1980-2019 | 37 | limit 36 to yr="1980 -Current" |
| language | 38 | 37 and (english or french or german).lg. |

Agricultural & Environmental Science Database (ProQuest)

| Set | Search |
| --- | --- |
| S11 | (MAINSUBJECT.EXACT("Humans") OR MAINSUBJECT.EXACT("Human biology") OR MAINSUBJECT.EXACT("Human impact") OR MAINSUBJECT.EXACT("Children & youth") OR MAINSUBJECT.EXACT("Adults") OR MAINSUBJECT.EXACT("Blood") OR MAINSUBJECT.EXACT("Bioaccumulation")) OR noft((human* OR men OR women OR adult* OR child* OR blood OR health)) |
| S12 | (MAINSUBJECT.EXACT("Lead poisoning") OR MAINSUBJECT.EXACT("Lead")) OR noft(((lead OR Pb) NEAR/3 (level* OR blood OR concentration* OR metal* OR poison* OR intoxication OR toxicity OR expos* OR hazard* OR pollut* OR contaminat* OR ingest*))) |
| S13 | (MAINSUBJECT.EXACT("Soil contamination") OR MAINSUBJECT.EXACT("Soil contamination")) OR noft(((lead OR Pb) NEAR/2 (soil* OR sediment* OR field* OR agricultur* OR ground OR earth OR land OR surface*))) |
| S14 | (MAINSUBJECT.EXACT("Bioremediation") OR MAINSUBJECT.EXACT("Soil remediation") OR MAINSUBJECT.EXACT("Phytoremediation") OR MAINSUBJECT.EXACT("Remediation")) OR noft((remediation OR abatement OR phytoremediation OR phytostabili*ation OR phytoextraction OR phytomediation OR phytovolatili*ation OR bioremediation OR vitrification)) OR noft(((abate* OR abating OR clean-up OR cleanup OR decontaminat* OR remedia* OR biochar*) NEAR/3 (soil* OR sediment*))) OR noft(((decreas* OR reduc* OR encapsulat* OR extract* OR flush* OR immobili* OR solidifi* OR stabili*) NEAR/3 (lead OR Pb))) |
| S15 | S11 AND S12 AND S13 AND S14 |
| S16 | S11 AND S12 AND S13 AND S14 Languages: Englisch; Deutsch; Französisch |

Web of Science (SCI-EXPANDED, SSCI) (Clarivate)

| Set | Search |
| --- | --- |
| # 1 | TS=((lead OR Pb) NEAR/2 (soil* OR sediment* OR field* OR agricultur* OR ground OR earth OR land OR surface*)) |
| # 2 | TS=((lead OR Pb) NEAR/3 (level* OR blood OR concentration* OR metal* OR poison* OR intoxication OR toxicity OR expos* OR hazard* OR pollut* OR contaminat* OR ingest*)) AND TS=(soil* OR sediment*) |
| # 3 | #2 OR #1 |
| # 4 | TS=(remediation OR abatement OR phytoremediation OR phytostabili*ation OR phytoextraction OR phytomediation OR phytovolatili*ation OR bioremediation OR vitrification) |
| # 5 | TS=((abate* OR abating OR clean-up OR cleanup OR decontaminat* OR remedia* OR biochar*) NEAR/3 (soil* OR sediment*)) |
| # 6 | TS=((decreas* OR reduc* OR encapsulat* OR extract* OR flush* OR immobili* OR solidifi* OR stabili*) NEAR/3 (lead OR Pb)) |
| # 7 | #6 OR #5 OR #4 |
| # 8 | #7 AND #3 |
| # 9 | TS=(human* OR men OR women OR adult* OR child* OR blood OR health) |
| # 10 | #9 AND #8 |
| # 12 | #7 AND #3 Refined by: RESEARCH AREAS: ( PHYSIOLOGY OR HEMATOLOGY OR SPORT SCIENCES OR BEHAVIORAL SCIENCES OR TOXICOLOGY OR GENERAL INTERNAL MEDICINE OR PUBLIC ENVIRONMENTAL OCCUPATIONAL HEALTH OR ONCOLOGY OR GASTROENTEROLOGY HEPATOLOGY OR BIOCHEMISTRY MOLECULAR BIOLOGY OR CARDIOVASCULAR SYSTEM CARDIOLOGY OR MICROBIOLOGY OR TROPICAL MEDICINE OR ANTHROPOLOGY OR LIFE SCIENCES BIOMEDICINE OTHER TOPICS OR RESEARCH EXPERIMENTAL MEDICINE OR SURGERY OR SOCIAL SCIENCES OTHER TOPICS OR SOCIOLOGY OR PEDIATRICS ) |
| # 11 | #7 AND #3 Refined by: WEB OF SCIENCE CATEGORIES: ( ENGINEERING BIOMEDICAL OR PHARMACOLOGY PHARMACY OR MEDICINE GENERAL INTERNAL OR TOXICOLOGY OR ONCOLOGY OR PUBLIC ENVIRONMENTAL OCCUPATIONAL HEALTH OR CARDIAC CARDIOVASCULAR SYSTEMS OR MEDICINE RESEARCH EXPERIMENTAL OR NEUROSCIENCES OR CELL BIOLOGY OR PEDIATRICS ) |
| # 13 | #12 OR #11 OR #10 |
| # 14 | #12 OR #11 OR #10 Refined by: LANGUAGES: ( ENGLISH OR GERMAN OR FRENCH ) |

Scopus (Elsevier)

| History Count | Search Terms |
| --- | --- |
| 1 | TITLE-ABS-KEY ( ( lead  OR  pb )  W/2  ( soil*  OR  sediment*  OR  field*  OR  agricultur*  OR  ground  OR  earth  OR  land  OR  surface* ) ) |
| 2 | ( TITLE-ABS-KEY ( ( lead  OR  pb )  W/2  ( level*  OR  blood  OR  concentration*  OR  metal*  OR  poison*  OR  intoxication  OR  toxicity  OR  expos*  OR  hazard*  OR  pollut*  OR  contaminat*  OR  ingest* ) )  AND  TITLE-ABS-KEY ( soil*  OR  sediment* ) ) |
| 4 | ( TITLE-ABS-KEY ( ( lead  OR  pb )  W/2  ( soil*  OR  sediment*  OR  field*  OR  agricultur*  OR  ground  OR  earth  OR  land  OR  surface* ) ) )  OR  ( ( TITLE-ABS-KEY ( ( lead  OR  pb )  W/2  ( level*  OR  blood  OR  concentration*  OR  metal*  OR  poison*  OR  intoxication  OR  toxicity  OR  expos*  OR  hazard*  OR  pollut*  OR  contaminat*  OR  ingest* ) )  AND  TITLE-ABS-KEY ( soil*  OR  sediment* ) ) ) |
| 5 | ( TITLE-ABS-KEY ( remediation  OR  abatement  OR  phytoremediation  OR  phytostabili*ation  OR  phytoextraction  OR  phytomediation  OR  phytovolatili*ation  OR  bioremediation  OR  vitrification )  OR  TITLE-ABS-KEY ( ( abate*  OR  abating  OR  clean-up  OR  cleanup  OR  decontaminat*  OR  remedia*  OR  biochar* )  W/3  ( soil*  OR  sediment* ) )  OR  TITLE-ABS-KEY ( ( decreas*  OR  reduc*  OR  encapsulat*  OR  extract*  OR  flush*  OR  immobili*  OR  solidifi*  OR  stabili* )  W/3  ( lead  OR  pb ) ) ) |
| 6 | ( ( TITLE-ABS-KEY ( ( lead  OR  pb )  W/2  ( soil*  OR  sediment*  OR  field*  OR  agricultur*  OR  ground  OR  earth  OR  land  OR  surface* ) ) )  OR  ( ( TITLE-ABS-KEY ( ( lead  OR  pb )  W/2  ( level*  OR  blood  OR  concentration*  OR  metal*  OR  poison*  OR  intoxication  OR  toxicity  OR  expos*  OR  hazard*  OR  pollut*  OR  contaminat*  OR  ingest* ) )  AND  TITLE-ABS-KEY ( soil*  OR  sediment* ) ) ) )  AND  ( ( TITLE-ABS-KEY ( remediation  OR  abatement  OR  phytoremediation  OR  phytostabili*ation  OR  phytoextraction  OR  phytomediation  OR  phytovolatili*ation  OR  bioremediation  OR  vitrification )  OR  TITLE-ABS-KEY ( ( abate*  OR  abating  OR  clean-up  OR  cleanup  OR  decontaminat*  OR  remedia*  OR  biochar* )  W/3  ( soil*  OR  sediment* ) )  OR  TITLE-ABS-KEY ( ( decreas*  OR  reduc*  OR  encapsulat*  OR  extract*  OR  flush*  OR  immobili*  OR  solidifi*  OR  stabili* )  W/3  ( lead  OR  pb ) ) ) )  ... |
| 12 | ( TITLE-ABS-KEY ( human*  OR  men  OR  women  OR  adult*  OR  child*  OR  blood  OR  health  OR  population  OR  medic* ) )  AND  ( ( ( TITLE-ABS-KEY ( ( lead  OR  pb )  W/2  ( soil*  OR  sediment*  OR  field*  OR  agricultur*  OR  ground  OR  earth  OR  land  OR  surface* ) ) )  OR  ( ( TITLE-ABS-KEY ( ( lead  OR  pb )  W/2  ( level*  OR  blood  OR  concentration*  OR  metal*  OR  poison*  OR  intoxication  OR  toxicity  OR  expos*  OR  hazard*  OR  pollut*  OR  contaminat*  OR  ingest* ) )  AND  TITLE-ABS-KEY ( soil*  OR  sediment* ) ) ) )  AND  ( ( TITLE-ABS-KEY ( remediation  OR  abatement  OR  phytoremediation  OR  phytostabili*ation  OR  phytoextraction  OR  phytomediation  OR  phytovolatili*ation  OR  bioremediation  OR  vitrification )  OR  TITLE-ABS-KEY ( ( abate*  OR  abating  OR  clean-up  OR  cleanup  OR  decontaminat*  OR  remedia*  OR  biochar* )  W/3  ( soil*  OR  sediment* ) )  OR  TITLE-ABS-KEY ( ( decreas*  OR  reduc*  OR  encapsulat*  OR  extract*  OR  flush*  OR  immobili*  OR  solidifi*  OR  stabili* )  W/3  ( lead  OR  pb ) ) ) ) )  ... |
| 11 | TITLE-ABS-KEY ( human*  OR  men  OR  women  OR  adult*  OR  child*  OR  blood  OR  health  OR  population  OR  medic* ) |
| 13 | ( TITLE-ABS-KEY ( human*  OR  men  OR  women  OR  adult*  OR  child*  OR  blood  OR  health  OR  population  OR  medic* ) )  AND  ( ( ( TITLE-ABS-KEY ( ( lead  OR  pb )  W/2  ( soil*  OR  sediment*  OR  field*  OR  agricultur*  OR  ground  OR  earth  OR  land  OR  surface* ) ) )  OR  ( ( TITLE-ABS-KEY ( ( lead  OR  pb )  W/2  ( level*  OR  blood  OR  concentration*  OR  metal*  OR  poison*  OR  intoxication  OR  toxicity  OR  expos*  OR  hazard*  OR  pollut*  OR  contaminat*  OR  ingest* ) )  AND  TITLE-ABS-KEY ( soil*  OR  sediment* ) ) ) )  AND  ( ( TITLE-ABS-KEY ( remediation  OR  abatement  OR  phytoremediation  OR  phytostabili*ation  OR  phytoextraction  OR  phytomediation  OR  phytovolatili*ation  OR  bioremediation  OR  vitrification )  OR  TITLE-ABS-KEY ( ( abate*  OR  abating  OR  clean-up  OR  cleanup  OR  decontaminat*  OR  remedia*  OR  biochar* )  W/3  ( soil*  OR  sediment* ) )  OR  TITLE-ABS-KEY ( ( decreas*  OR  reduc*  OR  encapsulat*  OR  extract*  OR  flush*  OR  immobili*  OR  solidifi*  OR  stabili* )  W/3  ( lead  OR  pb ) ) ) ) )  AND  ( LIMIT-TO ( LANGUAGE ,  "English" )  OR  LIMIT-TO ( LANGUAGE ,  "French" )  OR  LIMIT-TO ( LANGUAGE ,  "German" ) )  ... |
| 14 | ( TITLE-ABS-KEY ( human*  OR  men  OR  women  OR  adult*  OR  child*  OR  blood  OR  health  OR  population  OR  medic* ) )  AND  ( ( ( TITLE-ABS-KEY ( ( lead  OR  pb )  W/2  ( soil*  OR  sediment*  OR  field*  OR  agricultur*  OR  ground  OR  earth  OR  land  OR  surface* ) ) )  OR  ( ( TITLE-ABS-KEY ( ( lead  OR  pb )  W/2  ( level*  OR  blood  OR  concentration*  OR  metal*  OR  poison*  OR  intoxication  OR  toxicity  OR  expos*  OR  hazard*  OR  pollut*  OR  contaminat*  OR  ingest* ) )  AND  TITLE-ABS-KEY ( soil*  OR  sediment* ) ) ) )  AND  ( ( TITLE-ABS-KEY ( remediation  OR  abatement  OR  phytoremediation  OR  phytostabili*ation  OR  phytoextraction  OR  phytomediation  OR  phytovolatili*ation  OR  bioremediation  OR  vitrification )  OR  TITLE-ABS-KEY ( ( abate*  OR  abating  OR  clean-up  OR  cleanup  OR  decontaminat*  OR  remedia*  OR  biochar* )  W/3  ( soil*  OR  sediment* ) )  OR  TITLE-ABS-KEY ( ( decreas*  OR  reduc*  OR  encapsulat*  OR  extract*  OR  flush*  OR  immobili*  OR  solidifi*  OR  stabili* )  W/3  ( lead  OR  pb ) ) ) ) )  AND  ( LIMIT-TO ( LANGUAGE ,  "English" )  OR  LIMIT-TO ( LANGUAGE ,  "French" )  OR  LIMIT-TO ( LANGUAGE ,  "German" ) )  AND  ( EXCLUDE ( PUBYEAR ,  1979 )  OR  EXCLUDE ( PUBYEAR ,  1978 )  OR  EXCLUDE ( PUBYEAR ,  1977 )  OR  EXCLUDE ( PUBYEAR ,  1976 )  OR  EXCLUDE ( PUBYEAR ,  1975 )  OR  EXCLUDE ( PUBYEAR ,  1974 )  OR  EXCLUDE ( PUBYEAR ,  1973 )  OR  EXCLUDE ( PUBYEAR ,  1972 ) )  ... |

**Website Searches**

U.S. Department of Housing and Urban Development <https://www.hud.gov/>

21. July 2019

| Term searched | Filter |
| --- | --- |
| soil | Adobe PDF |
| lead | Adobe PDF |
| Soil AND lead | Adobe PDF |
| abatement | Adobe PDF |
| Soil AND lead AND abatement | Adobe PDF |
| soil AND abatement AND (lead OR plumb OR pb) | Adobe PDF |
| Soil AND abatement - paint AND (lead OR plumb OR pb) | Adobe PDF |

- Only searched July 2019, website inaccessible

WHO - ICTRP Search Portal <https://apps.who.int/trialsearch/>

26 April 2021

| Term searched |
| --- |
| soil |
| lead |
| Soil AND lead |
| abatement |
| Soil AND lead AND abatement |
| soil AND abatement AND (lead OR plumb OR pb) |
| Soil AND abatement - paint AND (lead OR plumb OR pb) NOT paint |
| remediation |

The Australian National Health and Medical Research Council https://www.nhmrc.gov.au/

13 April 2021

| Term searched |
| --- |
| Soil |
| Lead |
| Soil AND lead |
| Abatement |
| Soil AND lead AND abatement |
| Soil AND abatement AND (lead OR plumb OR pb |
| Soil AND abatement – paint AND (lead OR plumb OR pb) |

Médecins sans Frontières - <https://www.msf.org/>

13 April 2021

| Term searched |
| --- |
| soil |
| lead |
| Soil AND lead |
| abatement |
| Soil AND lead AND abatement |
| soil AND abatement AND (lead OR plumb OR pb) |
| Soil AND abatement - paint AND (lead OR plumb OR pb) NOT paint |
| Soil AND (lead OR plumb OR pb) AND abatement NOT paint |
| Update Search:  Remediation |

Health Canada - <https://www.canada.ca/en/health-canada.html>

13 April 2021

| Term searched |
| --- |
| soil |
| lead |
| Soil AND lead |
| abatement |
| Soil AND lead AND abatement |
| Soil abatement (lead OR plumb OR pb) |
| Soil AND abatement NOT paint (lead OR plumb OR pb) |
| Soil lead abatement NOT paint |
| Original Search: Soil lead abatement NOT paint OR groundwater  Update Search: Soil lead abatement NOT (paint OR groundwater) |
| soil AND (lead OR pb OR plumb) AND abatement AND human |
| soil AND (lead OR pb OR plumb) AND abatement |

National Service Center for Environmental Publications (NSCEP) <https://nepis.epa.gov>

13 April 2021

Search terms: Lead soil abatement Remediation human blood

# Appendix B. List of Excluded Studies and Reasons of Exclusion

***a) Ineligible study design (32)***

1. GROVES, B. D. & WEIDNER, C. G. 1993. The expanding role of the industrial hygienist: Controlling both public and worker exposures during remediation of lead- and mercury-contaminated soil at 130 residential properties. Applied Occupational and Environmental Hygiene, 8, 984-987.

2. JEZ, E. & LESTAN, D. 2015. Prediction of blood lead levels in children before and after remediation of soil samples in the upper Meza Valley, Slovenia. Journal of Hazardous Materials, 296, 138-146.

3. LAIDLAW, M. A. S., FILIPPELLI, G. M., BROWN, S., PAZ-FERREIRO, J., REICHMAN, S. M., NETHERWAY, P., TRUSKEWYCZ, A., BALL, A. S. & MIELKE, H. W. 2017. Case studies and evidence-based approaches to addressing urban soil lead contamination. Applied Geochemistry, 83, 14-30.

4. LEWANDOWSKI, T. A. & FORSLUND, B. L. 1994. Comparison of IEUBK model predictions and actual blood lead values at a former battery recycling site. Environmental Geochemistry & Health, 16, 217-22.

5. LORENZANA, R. M., TROAST, R., MASTRIANO, M., FOLLANSBEE, M. H. & DIAMOND, G. L. 2003. Lead intervention and pediatric blood lead levels at hazardous waste sites. Journal of Toxicology & Environmental Health Part A, 66, 871-93.

6. LOUEKARI, K., MROUEH, U. M., MAIDELL-MUNSTER, L., VALKONEN, S., TUOMI, T. & SAVOLAINEN, K. 2004. Reducing the risks of children living near the site of a former lead smeltery. Science of the Total Environment, 319, 65-75.

7. MAISONET, M., BOVE, F. J. & KAYE, W. E. 1997. A case-control study to determine risk factors for elevated blood lead levels in children, Idaho. Toxicology & Industrial Health, 13, 67-72.

8. NATIONAL SERVICE CENTER FOR ENVIRONMENTAL PUBLICATIONS (NSCEP). 1995. Review of studies addressing lead abatement effectiveness [Online].

9. NATIONAL SERVICE CENTER FOR ENVIRONMENTAL PUBLICATIONS (NSCEP). 1995. Urban soil lead abatement demonstration project. EPA Integrated Report [Online].

10. ROSEN, J. F. 2003. A critical evaluation of public health programs at the Bunker Hill Superfund site. Science of the Total Environment, 303, 15-23.

11. RYAN, J. A., SCHECKEL, K. G., BERTI, W. R., BROWN, S. L., CASTEEL, S. W., CHANEY, R. L., HALLFRISCH, J., DOOLAN, M., GREVATT, P., MADDALONI, M. & MOSBY, D. 2004. Reducing children's risk from lead in soil. Environmental Science & Technology, 38, 18A-24A.

12. SHELDRAKE, S. & STIFELMAN, M. 2003. A case study of lead contamination cleanup effectiveness at Bunker Hill. Sci Total Environ, 303, 105-23.

13. THOMSON, C. F., POPPE, B., CLARK, C. S., RICE, C. H. & LINZ, D. 1992. Development and Implementation of a Safety and Health Program for Employees Involved with Residential Soil and Dust Lead Abatement and Monitoring. Applied Occupational and Environmental Hygiene, 7, 398.

14. TIRIMA, S., BARTREM, C., VON LINDERN, I., VON BRAUN, M., LIND, D., ANKA, S. M. & ABDULLAHI, A. 2016. Environmental Remediation to Address Childhood Lead Poisoning Epidemic due to Artisanal Gold Mining in Zamfara, Nigeria. Environ Health Perspect, 124, 1471-8.

15. TSUJI, J. S. & SERL, K. M. 1996. Current uses of the EPA lead model to assess health risk and action levels for soil. Environmental Geochemistry & Health, 18, 25-33.

16. VAOULI, L. & POMALES-SCHICK, A. 2015. Using soilSHOPs to Reduce Community Exposures to Lead in Soils. Journal of Environmental Health, 78, 24-27.

17. WANG, L., YANG, D., LI, Z., FU, Y., LIU, X., BROOKES, P. C. & XU, J. 2019. A comprehensive mitigation strategy for heavy metal contamination of farmland around mining areas - Screening of low accumulated cultivars, soil remediation and risk assessment. Environmental Pollution, 245, 820-828.

18. WESOLOWSKI, J. J., FLESSEL, C. P., TWISS, S., STANLEY, R. L., KNIGHT, M. W., COLEMAN, G. C. & DEGARMO, T. E. 1979. The identification and elimination of a potential lead hazard in an urban park. Archives of environmental health, 34, 413-418.

19. Indian Geotechnical Conference,IGC 2018 2021;88(): 2021

20. PELLETIER, D.; SACKS, V. P.; SORENSEN, M.; MAGAR, V. Review of Remediation Goals at Contaminated Sediment Sites in the United States. Integr 2019;15(5):772-782, 2019

21. GAILEY, A. D.; SCHACHTER, A. E.; EGENDORF, S. P.; MIELKE, H. W. Quantifying soil contamination and identifying interventions to limit health risks. Curr. Probl. Pediatr. Adolesc. Health Care 2020;50(1):10, 2020

22. SOUZA LILIAN RODRIGUES, ROSA; POMAROLLI LUIZA, CAROLINA; DA VEIGA MÁRCIA ANDREIA MESQUITA, SILVA. From classic methodologies to application of nanomaterials for soil remediation: an integrated view of methods for decontamination of toxic metal(oid)s. Environmental Science and Pollution Research International 2020;27(10):10205-10227, 2020

23. KUMAR, A.; CABRAL-PINTO, M.; CHATURVEDI, A. K.; SHABNAM, A. A.; SUBRAHMANYAM, G.; MONDAL, R.; GUPTA, D. K.; MALYAN, S. K.; KUMAR, S. S.; KHAN, S. A.; YADAV, K. K. Lead toxicity: Health hazards, influence on food Chain, and sustainable remediation approaches. International Journal of Environmental Research and Public Health 2020;17(7): 2020

24. HU, B.; SHAO, S.; NI, H.; FU, Z.; HU, L.; ZHOU, Y.; MIN, X.; SHE, S.; CHEN, S.; HUANG, M.; ZHOU, L.; LI, Y.; SHI, Z. Current status, spatial features, health risks, and potential driving factors of soil heavy metal pollution in China at province level. Environmental Pollution 2020;266():2020

25. ZHANG, Y.; O'CONNOR, D.; XU, W.; HOU, D. Blood lead levels among Chinese children: The shifting influence of industry, traffic, and e-waste over three decades. Environment International 2020;135(): 2020

26. 4th International Conference on Biological Sciences and Biotechnology. 2019;305(): 2019

27. LEVIN, R.; VIEIRA, C. L. Z.; MORDARSKI, D. C.; ROSENBAUM, M. H. Lead seasonality in humans, animals, and the natural environment. Environ. Res. 2020;180():11, 2020

28. HAYNES, H. M.; TAYLOR, K. G.; ROTHWELL, J.; BYRNE, P. Characterisation of road-dust sediment in urban systems: a review of a global challenge. J. Soils Sediments 2020;20(12):4194-4217, 2020

29. NUSSBAUMER-STREIT, B.; MAYR, V.; DOBRESCU, A. I.; WAGNER, G.; CHAPMAN, A.; PFADENHAUER, L. M.; LOHNER, S.; LHACHIMI, S. K.; BUSERT, L. K.; GARTLEHNER, G. Household interventions for secondary prevention of domestic lead exposure in children. Cochrane Database Syst Rev 2020;10():CD006047, 2020

30. UDIBA, U. U.; AKPAN, E. R.; ANTAI, E. E. Soil Lead Concentrations in Dareta Village, Zamfara, Nigeria. J 2019;9(23):190910, 2019

31. MOHAMMED, M. A.; MOHD YUNUS, N. Z.; HEZMI, M. A.; ABANG HASBOLLAH, D. Z.; AS, A. RASHID. Ground improvement and its role in carbon dioxide reduction: a review

Environ Sci Pollut Res Int 2021;14():14, 2021

32. AMIN CHOWDHURY, K. I.; NURUNNAHAR, S.; KABIR, M. L.; ISLAM, M. T.; BAKER, M.; ISLAM, M. S.; RAHMAN, M.; HASAN, M. A.; SIKDER, A.; KWONG, L. H.; BINKHORST, G. K.; NASH, E.; KEITH, J.; MCCARTOR, A.; LUBY, S. P.; FORSYTH, J. E. Child lead exposure near abandoned lead acid battery recycling sites in a residential community in Bangladesh: risk factors and the impact of soil remediation on blood lead levels. Environ Res 2021;():110689, 2021

***b) Ineligible document type (1)***

1. SCHLENKER, T. L. 1993. Soil abatement and lead levels in children. JAMA, 270, 829-30.

***c) Ineligible study population (2)***

1. HYNES, H. P., MAXFIELD, R., CARROLL, P. & HILLGER, R. 2001. Dorchester Lead-Safe Yard project: a pilot program to demonstrate low-cost, on-site techniques to reduce exposure to lead-contaminated soil. Journal of Urban Health, 78, 199-211.

2. WANG, L.; YANG, D.; LI, Z.; FU, Y.; LIU, X.; BROOKES, P. C.; XU, J. A comprehensive mitigation strategy for heavy metal contamination of farmland around mining areas – Screening of low accumulated cultivars, soil remediation and risk assessment. Environmental Pollution 2019;245():820-828, 2019

***d) Ineligible intervention (16)***

1. BORELAND, F., LESJAK, M. & LYLE, D. 2009. Evaluation of home lead remediation in an Australian mining community. Science of the Total Environment, 408, 202-208.

2. DE FREITAS, C. U., DE CAPITANI, E. M., GOUVEIA, N., SIMONETTI, M. H., DE PAULA E SILVA, M. R., KIRA, C. S., SAKUMA, A. M., DE FÁTIMA HENRIQUES CARVALHO, M., DURAN, M. C., TIGLEA, P. & DE ABREU, M. H. 2007. Lead exposure in an urban community: Investigation of risk factors and assessment of the impact of lead abatement measures. Environmental Research, 103, 338-344.

3. ERICSON, B. 2014. Mitigation of acute lead exposures in Dong Mai Village, Vietnam. Blacksmith Institute.

4. ERICSON, B., DUONG, T. T., KEITH, J., NGUYEN, T. C., HAVENS, D., DANIELL, W., KARR, C. J., NGOC HAI, D., VAN TUNG, L., THI NHI HA, T., WILSON, B., HANRAHAN, D., CROTEAU, G. & PATRICK TAYLOR, M. 2018. Improving human health outcomes with a low-cost intervention to reduce exposures from lead acid battery recycling: Dong Mai, Vietnam. Environmental Research, 161, 181-187.

5. GLOTZER, D. E., WEITZMAN, M., ASCHENGRAU, A. & FREEDBERG, K. A. 1997. Economic evaluation of environmental interventions for low-level childhood lead poisoning. Ambulatory Child Health, 3, 255-267.

6. GOULET, L., GAUDREAU, J. & MESSIER, A. 1996. Results of a lead decontamination program. Arch Environ Health, 51, 68-72.

7. LALOR, G., RATTRAY, R., VUTCHKOV, M., CAMPBELL, B. & LEWIS-BELL, K. 2001. Blood lead levels in Jamaican school children. Science of the Total Environment, 269, 171-181.

8. LANGLOIS, P., SMITH, L., FLEMING, S., GOULD, R., GOEL, V. & GIBSON, B. 1996. Blood lead levels in Toronto children and abatement of lead-contaminated soil and house dust. Archives of Environmental Health, 51, 59-67.

9. MAYNARD, E., THOMAS, R., SIMON, D., PHIPPS, C., WARD, C. & CALDER, I. 2003. An evaluation of recent blood lead levels in Port Pirie, South Australia. Science of the Total Environment, 303, 25-33.

10. RUBIO-ANDRADE, M., VALDES-PEREZGASGA, F., ALONSO, J., ROSADO, J. L., CEBRIAN, M. E. & GARCIA-VARGAS, G. G. 2011. Follow-up study on lead exposure in children living in a smelter community in northern Mexico. Environmental Health: A Global Access Science Source, 10, 66.

11. SCHOOF, R. A., JOHNSON, D. L., HANDZIUK, E. R., LANDINGHAM, C. V., FELDPAUSCH, A. M., GALLAGHER, A. E., DELL, L. D. & KEPHART, A. 2016. Assessment of blood lead level declines in an area of historical mining with a holistic remediation and abatement program. Environ Res, 150, 582-591.

12. SHAO, L., ZHANG, L. & ZHEN, Z. 2017. Interrupted time series analysis of children's blood lead levels: A case study of lead hazard control program in Syracuse, New York. PLoS ONE [Electronic Resource], 12, e0171778.

13. AELION, C. M.; DAVIS, H. T. Blood lead levels in children in urban and rural areas: Using multilevel modeling to investigate impacts of gender, race, poverty, and the environment

Science of the Total Environment 2019;694(): 2019

14. AHMAD, I.; KHAN, B.; ASAD, N.; MIAN, I. A.; JAMIL, M. Traffic-related lead pollution in roadside soils and plants in Khyber Pakhtunkhwa, Pakistan: implications for human health. International Journal of Environmental Science and Technology 2019;16(12):8015-8022, 2019

15. MIELKE, H. W.; GONZALES, C. R.; POWELL, E. T. Curtailing Lead Aerosols: Effects of Primary Prevention on Declining Soil Lead and Children's Blood Lead in Metropolitan New Orleans. Int J Environ Res Public Health 2019;16(12):12, 2019

16. BINESHPOUR, M.; PAYANDEH, K.; NAZARPOUR, A.; SABZALIPOUR, S. Status, source, human health risk assessment of potential toxic elements (PTEs), and Pb isotope characteristics in urban surface soil, case study: Arak city, Iran, Environ. Geochem. Health ;():20

***e) Fulltext not retrievable (10)***

1. ABOU-ZEID, A. H. & RICCI, P. F. 2008. Public Health Risk Assessment: An Egyptian Study of Secondary Lead Smelting and Associated Risks. International Journal of Environment and Health, 2, 147.

2. ANONYMOUS 1992. Toxic substances: Federal programs do not fully address some lead exposure issues. UNITED STATES GENERAL ACCOUNTING OFFICE, P.O. BOX 6015, GAITHERSBURG, MD 20877 (USA).

3. ANONYMOUS 2003. Composted biosolids bind lead in soil, reducing danger of poisoning. Journal of Environmental Health, 65, 36-36.

4. DOR, F. & DENYS, S. 2011. Combining environmental and health evaluation is essential to optimize the management of contaminated sites: A case study of Saint-Laurent-le-Minier. Environnement Risques & Sante, 10, 323-330.

5. EIDSON, M. & TOLLESTRUP, K. 1995. Blood lead levels and remediation of an abandoned smelter site. Journal of Environmental Health, 57, 8-14.

6. MADDALONI, M. 2013. State of the science: New recommendations on elevated Pb blood level and implications to lead soil remediation. Abstracts of Papers of the American Chemical Society, 246, 1.

7. MIELKE, H. W., ADAMS, J. E., HUFF, B., PEPERSACK, J., REAGAN, P. L., STOPPEL, D. & MIELKE JR, P. W. Dust control as a means of reducing inner-city childhood Pb exposure. 1994 Columbia, MO, United States. Publ by Univ of Missouri, 121-128.

8. TAYLOR, J. R. & FORSLUND, B. L. 1991. Environmental Impacts on Blood-Lead Levels in the Vicinity of a Former Battery Recycling Plant. EPA/Univ of Missouri/et al Trace Subst in Environ Health 25th Conf, Columbia, MO, 105.

9. TAYLOR, J. R. & FORSLUND, B. L. Environmental impacts on blood-lead levels in the vicinity of a former battery recycling plant. 1994 Columbia, MO, United States. Publ by Univ of Missouri, 105-119.

10. WEITZMAN, M., ASCHENGRAU, A. & BELLINGER, D. 1993. SOIL ABATEMENT AND LEAD LEVELS IN CHILDREN - REPLY. Jama-Journal of the American Medical Association, 270, 829-830.

***f) Duplicate (4)***

1. MIELKE, H. W., ADAMS, J. E., HUFF, B., PEPERSACK, J., REAGAN, P. L., STOPPEL, D. & MIELKE, P. W. 1991. Dust Control as a Means of Reducing Inner-City Childhood Pb Exposure. EPA/Univ of Missouri/et al Trace Subst in Environ Health 25th Conf, Columbia, MO, 121.

2. NATIONAL SERVICE CENTER FOR ENVIRONMENTAL PUBLICATIONS (NSCEP). 1996. Urban soil lead abatement demonstration project: Volume I: EPA Integrated Report [Online].

3. NATIONAL SERVICE CENTER FOR ENVIRONMENTAL PUBLICATIONS (NSCEP). 1998. Review of Studies Addressing Lead Abatement Effectiveness: Updated Edition [Online].

4. WEITZMAN, M. 1993. Lead-contaminated soil abatement and urban children's blood lead levels. Weitzman M, Aschengrau A, Bellinger D, et al. JAMA 1993; 269:1647-54. The Journal of Pediatrics, 123, 832

# Appendix C. Certainty of evidence ratings

### Soil remediation with co-intervention versus co-intervention for preventing lead exposure

| **Population**: children  **Setting**: general population  **Intervention**: soil remediation with co-interventions  **Comparison**: only co-intervention | | | |
| --- | --- | --- | --- |
| Outcomes | Impact | №. of participants (studies) | Certainty of the evidence (GRADE) |
| BLL decrease measured as μg/dL Follow-up: 11 months to 1 year | The adjusted mean differences in BLL was -0.80 μg/dL (95% CI: 0.45, -2.05) in one study (11 months follow-up) and -0.05 μg/dL (95% CI -0.12, 0.03) in another (one year follow-up) | 511 (2 RCTs*) | ⨁⨁◯◯ LOW^a, b^ |

^*^ Based on studies by Weitzman et al., 1993[26] and Farrell et al., 1998[27]

^a^high risk of bias because of a high attrition rate (55%) in the larger of the two studies (Farrell et al. 1998)

^b^the confidence intervals cross the null and encompass values that could be considered an appreciable benefit

### Soil remediation versus no intervention for preventing lead exposure

| **Population**: children  **Setting**: general population  **Intervention**: soil remediation  **Comparison**: no intervention | | | |
| --- | --- | --- | --- |
| **Outcomes** | **Impact** | **№. of participants (studies)** | **Certainty of the evidence (GRADE)** |
| BLL decrease measured as μg/dL Follow-up 1 year | The mean difference in BLL from baseline to follow-up was higher in the intervention group (-2.5 μg/dL, p<0.0001) compared to the control group (-0.4 μg/dL, p<0.0001). 95% CIs were not reported. | 1425 (1 observational study*) | ⨁⨁◯◯ LOW^a, b^ |

*Abbreviations:* BLL = blood lead levels; CI = confidence interval; No, number of participants

^*^ Based on the study by von Lindern et al., 2003

^a^non-randomized study, BLL levels and other characteristics were different at baseline in the intervention and control group

^b^participants are children under the age of 9 years
